# An ICF-based interdisciplinary case management improves functioning, participation, and well-being for people with disabilities in community rehabilitation: a protocol for a randomised controlled trial

**DOI:** 10.3389/fresc.2026.1745671

**Published:** 2026-03-17

**Authors:** Chi Keung Yeung, Ka Man Cheng, Ping Kin Wong, Shui Sang Wong, Hon Yee Cheng, Wai Yee Cheung, Wai Yan Wong, Choi Wa Ling, Chi Chung Lee

**Affiliations:** 1 Yang Memorial Methodist Social Service, Hong Kong, Hong Kong SAR, China; 2 School of Health Sciences, Faculty of Medicine, Nursing, Midwifery and Health Sciences, The University of Notre Dame Australia, Sydney, NSW, Australia; 3 Department of Construction and Quality Management, School of Science and Technology, Hong Kong Metropolitan University, Hong Kong, Hong Kong SAR, China

**Keywords:** case management, community-based rehabilitation, interdisciplinary collaboration, international classification of functioning disability and health, people with disabilities, person-centred care, randomised control trial, social participation

## Abstract

**Background:**

Community-delivered rehabilitation (CDR) aims to empower people with disabilities and promote their social inclusion. However, conventional multidisciplinary approaches often lack integration and personalised coordination. The International Classification of Functioning, Disability and Health (ICF) offers a structured framework that facilitates interdisciplinary collaboration, case management and person-centred care. This trial aims to evaluate the effectiveness of an ICF-based interdisciplinary case management model in improving functioning, participation, and subjective well-being among adults with physical disabilities receiving home-based community rehabilitation, compared with usual multidisciplinary care.

**Methods:**

This is a six-month, single-blind, randomised controlled trial. Sixty participants will be recruited and randomly assigned via block randomisation to either an intervention group receiving structured ICF-based interdisciplinary case management or a control group receiving usual multidisciplinary care. The inclusion criteria were adults with physical disabilities eligible for CDR; exclusion criteria included severe cognitive impairment or inability to provide informed consent. The intervention consists of ICF-based assessments using the ICF Rehabilitation Set and Minimal Set of Environmental Factors, collaborative goal setting, monthly interdisciplinary case conferences, and coordinated care planning. The primary outcomes are functioning (WHO Disability Assessment Schedule 2.0, 36-item version) and well-being (Satisfaction with Life Scale). The secondary outcomes are changes in ICF qualifiers and therapist-related clinical outcomes (Australian Therapy Outcome Measures). Outcomes will be assessed at baseline and six months. Data will be analysed via intention-to-treat principles and generalised linear mixed models (GLMMs). Recruitment began in May 2025 and is expected to be completed by December 2025, with follow-up concluded by July 2026.

**Discussion:**

This trial will generate empirical evidence on the use of the ICF as a framework for structured interdisciplinary case management in CDR. The findings may inform service planning, interprofessional training, and policy development to promote holistic, person-centred disability care.

**Clinical Trial Registration:** https://www.chictr.org.cn/hvshowprojectEN.html?id=284308&v=1.0.

## 1 Introduction

Many people with disabilities (PWDs) face significant challenges in regaining independence, social participation and quality of life. The challenges are imposed not only by the limitations in physical capacity, but also by family members overprotecting individuals with disabilities by performing tasks for them, inadvertently limiting their autonomy, or societal attitudes may exclude them due to physical or social discrimination. Community-based rehabilitation (CBR) represents a paradigm shift in healthcare, prioritising the empowerment of PWD and fostering their social inclusion within their own community (1). Because it is implemented in more than 90 countries worldwide, CBR has arisen as a holistic approach to address the above challenges by enabling PWD to fully participate in the community (2, 3). The role of CBR is to empower PWD and their families by promoting, supporting and facilitating their active involvement in issues that affect their lives (4, 5).

Over the past four decades, the concept of CBR has evolved substantially. Originally introduced by the World Health Organization (WHO) in 1976 as a strategy to deliver rehabilitation services at community level, CBR initially focused on providing basic rehabilitation techniques in resource-limited settings (6). However, the conceptual framework of CBR has shifted towards a broader rights-based, multisectoral approach known as community-based inclusive development (CBID), which emphasises empowerment, advocacy, social inclusion, and equalisation of opportunities rather than direct service provision. Within this evolution, health-related rehabilitation delivered in community settings is more appropriately described as community-delivered rehabilitation (CDR), integrated within the health system and complementing institution-based services.

The International Classification of Functioning, Disability, and Health (ICF), developed by the WHO in 2001, offers a biopsychosocial model that integrates medical and social perspectives on health and disability. Unlike the traditional medical model, which focuses primarily on impairments, the ICF framework emphasises the functioning of a person across different components, including body functions, body structures, activities and participation, and contextual factors. The latter includes environmental facilitators, barriers and personal characteristics, thereby integrating medical and social dimensions of disability (7). It considers the interaction between the health conditions of PWD and the contextual factors that influence how PWD function and participate in their community, which, in turn, promotes a scientific understanding of functioning and disability. This comprehensive framework aligns with the principle of CDR, which aims to enable PWD to achieve and maintain optimal functioning by addressing the medical, psychosocial, economic and environmental aspects of rehabilitation.

It has been proposed that an interdisciplinary approach designed to optimise functioning in various domains provides benefits to PWD (8). Traditional multidisciplinary approaches, where different professionals work in parallel with limited integration of their interventions, often suffer from fragmentation and a lack of seamless coordination, potentially hindering the delivery of truly person-centred care (9). Interdisciplinary case management is a more personalised approach where different professionals collaborate with service users to assess their needs, set rehabilitation goals and ensure that the care is cohesive, goal-oriented and aligned with the unique functional and contextual needs (10, 11).

The ICF provides a framework and common language to promote interdisciplinary collaboration. Studies have demonstrated that ICF application enhances care coordination and interdisciplinary collaboration and provides a common language for describing and addressing disability (12, 13). Furthermore, an ICF-based assessment and intervention planning model specifically for community rehabilitation settings has been proposed, incorporating structured prioritisation mechanisms to guide case management in complex community contexts (6). Integrating the ICF into case management can enhance interprofessional learning by promoting a multidimensional perspective of an individual's health concerns (14, 15).

However, the focus on studying the clinical outcomes of the application of the ICF framework in interdisciplinary case management, particularly for individuals with physical disabilities, remains underexplored. Emerging research has shown that the clinical application of the ICF model in stroke survivors, including the use of the ICF core set in the assessment and goal setting, by a single rehabilitation discipline such as physiotherapy and occupational therapy, can improve patient outcomes (16, 17). Recently, Wong et al. (18) investigated an 8–12-week community-based multidisciplinary rehabilitation programme for poststroke patients in Hong Kong. The multidisciplinary team, composed of physiotherapists, occupational therapists and speech therapists, was responsible for offering different modules in ICF activities and participation domains. Each of the disciplines set goals and developed training content, emphasising resuming life roles and community reintegration. They revealed that the participants in their study showed significant improvements in all aspects of body functions, activities and participation, which also predict patients' satisfaction. The above studies suggest that the clinical application of ICF by a single professional or a multidisciplinary team could improve clinical outcomes. Studies have also suggested that an interdisciplinary team approach in rehabilitation could enhance teamwork and team effectiveness (19) and promote clinical outcomes (20) compared with usual care.

Therefore, it is reasonable to hypothesise that applying the ICF model in an interdisciplinary rehabilitation programme would be even more effective than a traditional multidisciplinary rehabilitation programme in enhancing functioning and facilitating community reintegration holistically in PWD, as an interdisciplinary approach allows for the integration of complementary expertise from different professionals to address the multifaceted needs of PWD. ICF-based approaches can further enhance interdisciplinary communication and reduce duplication of efforts among rehabilitation professionals, which in turn improves cost-effectiveness (21). Usual multidisciplinary care provides a relevant and pragmatic comparator, reflecting current community rehabilitation service structures. This allows us to evaluate the added value of an ICF-based Interdisciplinary case management (ICFIDCM) model, which is hypothesised to offer more coordinated, goal-oriented, and integrated care, potentially better addressing the complex, interactional needs of PWD (22).

Despite the theoretical and practical alignment between CDR and the ICF, several important gaps remain in both evidence and practice within the broader CBR literature. Many CBR programmes continue to focus predominantly on impairments and activity limitations, often giving less systematic attention to participation and the contextual factors that are central to the ICF model (5, 12, 23). This limited integration restricts the ability of CBR to fully realise its potential in promoting holistic, person-centred outcomes for PWD (13, 24).

Additionally, the application of the ICF framework in CDR programs varies widely across programs in regard to assessment, goal setting, and outcome evaluation (25, 26). The inconsistent application of ICF-based approaches across different settings creates difficulties when trying to compare results between locations and establish general findings about ICF-based intervention success (18). Another persistent gap is the underutilisation of interdisciplinary case management in community settings. While the benefits of interdisciplinary collaboration are well recognised in the literature (19, 27), including improved outcomes and cost-effectiveness in community-based interventions (20), practical implementation remains limited, often due to resource constraints, a lack of training, and systemic barriers (28).

In addition, while different core sets, e.g., the ICF Rehabilitation Set and Minimal Set of Environmental Factors, were developed originally for clinical use, their application is increasingly extending into broader statistical and monitoring contexts (29, 30). The absence of research on core set implementation in interdisciplinary case management for community rehabilitation requires studies that assess both clinical results, practicality, and usefulness of these tools for standardised care coordination (31, 32).

Perhaps most critically, the current research on ICF-based interventions in CDR relies mainly on observational and quasi-experimental studies (16, 18, 23, 33). This means that the current literature lacks sufficient high-quality randomised controlled trials (RCTs) that study the effects of structured ICFIDCM models on functioning, participation, and well-being in actual community settings (34).

The present study design aims to address the above knowledge gaps that exist in current evidence. It evaluates an ICFIDCM model within CDR services to measure its effects on the functioning, participation, and well-being of PWD. It uses ICF-based assessment tools and collaborative goal setting to deliver person-centred care while evaluating ICFIDCM against traditional multidisciplinary care. In doing so, this research aims to establish strong evidence that will help develop optimal CDR practices.

## 2 Methods and analysis

### 2.1 Research aim

The trial aims to evaluate the effectiveness of the ICFIDCM compared with that of usual multidisciplinary care in improving the functioning and well-being of people with disabilities who receive home-based community rehabilitation. Two primary outcome indicators, which are both patient-reported outcomes (PROs), are functioning (World Health Organization Disability Assessment Schedule 2.0, WHODAS 2.0, 36-item version) and well-being (Satisfaction with Life Scale, SWLS). Two secondary outcome indicators, therapist-reported outcomes (TROs), are changes in ICF qualifiers and Australian Therapy Outcome Measures (AusTOMs). We hypothesised that, compared with the usual care control group, participants receiving the ICFIDCM would demonstrate greater improvements from baseline to six months in terms of functioning (lower WHODAS 2.0 scores) and subjective well-being (higher SWLS scores). For the secondary outcome, compared with the control group, we expect to see greater pre-post improvements in therapist-rated functioning (improved ICF qualifier scores) and in therapist-rated clinical outcomes (higher AusTOMs scores) among participants in the intervention group.

### 2.2 Trial design

A six-month parallel group randomised controlled trial (RCT) will be conducted. PWDs newly referred to and eligible for the Home Care Service for Persons with Severe Disabilities (HCS), which provides home-based rehabilitation services, will be recruited. After providing informed consent and completing baseline assessments, participants will be randomly allocated to either the treatment group (ICFIDCM) or the control group (usual multidisciplinary case management). This study was registered with the Chinese Clinical Trial Registry (https://www.chictr.org.cn): (ChiCTR2500109152) and approved by the Research Ethics Committee of Hong Kong Metropolitan University (HE-SF2025/27). The study protocols followed the SPIRIT guidelines.

#### 2.2.1 Participants

##### 2.2.1.1 Inclusion criteria

Adults aged ≥18 years with diagnosed physical disabilities who are newly referred to and eligible for HCS, who are medically stable, and who are able to provide written consent.

##### 2.2.1.2 Exclusion criteria

Individuals with acute or unstable medical conditions requiring hospitalisation, terminal illnesses with a prognosis of less than six months, psychological disorders or severe mental illness.

Individuals with severe cognitive impairments, e.g., those diagnosed with severe dementia, intellectual disability, or other conditions preventing meaningful participation in the study.

### 2.3 Sample size determination

The primary outcomes of this study are PROs of functioning and well-being (WHODAS 2.0 and SWLS). We identified Hussain et al. (2025), who reported a between-group effect size of d = 0.8 on WHODAS 2.0 scores at the 20-week follow-up in a community-based, culturally adapted problem management intervention trial for people with disability (35). We use this study to inform our sample size calculation since the follow-up duration and outcome measure closely align with our six-month endpoint. Although Hussain et al. (2025) used the 12-item WHODAS 2.0 version, psychometric studies have demonstrated high concordance between the 12- and 36-item versions (correlation coefficients of *r* ≥ 0.915 between total scores on these two versions). This strong agreement supports the application of an effect size derived from the 12-item version to the 36-item WHODAS 2.0.

Using G*Power (G*Power 3, version 3.1.9.6) (36), with a α level of 0.05 (two-tailed), a power of 0.80, and an effect size of 0.8 as reported, a minimum sample size of approximately 26 participants per group (a total of 52 participants) was determined. Assuming a 15% attrition rate (18), the study will recruit a total of 60 participants who are PWD, newly referred and eligible service users of home care services. Considering that approximately 120–160 new case referrals are received per year, recruitment will take a minimum of 6 months.

### 2.4 Recruitment and consent procedures

Participants will be recruited from HCS operated by the Yang Memorial Methodist Social Service (YMMSS). New service users are identified through the service referral system. Trained social workers (case managers) approach potential participants, explain the study and obtain written informed consent at the participant's home. Written informed consent will be stored safely in the main office of the home care service teams.

Participants may withdraw from the study at any point without consequences. Dropouts will be recorded if a participant (a) withdraws consent or (b) dies before the follow-up assessment. Withdrawn cases will remain in the intention-to-treat analysis using their most recent available data. No dropout cases will be recruited.

### 2.5 Randomisation method

A computer-generated block randomisation sequence will be created in advance via Microsoft Excel by the secondary investigator (SI), who is not involved in participant recruitment or eligibility screening. This approach ensures equal allocation (1:1) to the intervention and control groups, and the sequence will remain unchanged throughout the study (37).

Each eligible service user referred to the home care service will be assigned a unique sequential referral number for administrative tracking and eligibility screening. Following eligibility screening, written informed consent and baseline assessment, allocation will be performed by the SI via the concealed randomisation sequence stored in a password-protected file. This file is only accessible only to the SI to ensure allocation concealment until the moment of assignment.

### 2.6 Participant blinding

The participants will not be informed whether they are receiving ICFIDCM or usual care (38, 39). Both groups will receive comparable regular professional contact and neutral communication to minimise the perception of group differences. The primary distinction between groups occurs through background care coordination mechanisms, with enhanced interprofessional collaboration and reasoning processes facilitated through the use of the ICF framework during case conferences in the intervention group vs. *ad hoc* coordination in the control group. Since participants are not directly involved in case conferences, group allocation differences will not apparently affect their service experience. As a result, participants remain unaware of whether they receive ICFIDCM or usual multidisciplinary care. In addition, while complete intervention provider (case manager, therapist and nurse) blinding is not feasible given the nature of the intervention, the research assistant responsible for collecting self-reported outcomes (WHODAS 2.0, SWLS) will remain blinded to group allocation to minimise bias.

### 2.7 Procedures

All professional staff involved in the study underwent structured training in applying the ICF framework in interdisciplinary case management and using outcome measures before the trial began. This training, delivered by the Principal Investigator (PI), an experienced ICF educator and practitioner in rehabilitation, covered foundational and advanced topics. The training included the biopsychosocial model, ICF classification and coding systems, qualifiers, interdisciplinary case management and collaborative goal setting, interprofessional case conferences, practical application of the ICF Rehabilitation Set and Minimal Set of Environmental Factors, WHODAS 2.0, SWLS, and AusTOMs. Supervision meetings will be held quarterly to reinforce fidelity and ensure consistent application of the ICF framework across the interdisciplinary team.

The participants will be PWD who are newly referred and eligible service users of the HCS, YMMSS. Recruitment will be conducted by the HCS Team. Eligible PWD will be identified from the service user referral system. A supervisor from the Home Care Service will assign trained social workers, who will act as case managers, to approach potential participants and confirm their eligibility to be enrolled in this study. Once the participants are confirmed to be eligible, the case managers provide verbal and written information about the study, answer any questions, and obtain written informed consent from the participants.

Upon consent, baseline assessments, which will take place during the initial intake, will be scheduled. Participants will complete baseline questionnaires and assessments, including the WHODAS 2.0 and SWLS. To minimise bias, a research assistant who is not involved in intervention delivery and is blinded to group allocation will be responsible for distributing, assisting (if needed), and collecting these self-administered questionnaires from participants (40). This approach ensures that the data collection for these key self-reported outcomes remains unbiased, as the assistant does not know which group the participant has been assigned to (41, 42). Assessments of TROs, such as the AusTOMs and ICF qualifiers using the ICF Rehabilitation Set and Minimal Set of Environmental Factors, will be conducted by the professional team.

Following the baseline assessment, participants will be randomly assigned at a 1:1 ratio to either the intervention group or the control group, as stated before. This study is single-blinded: participants will not be informed of their group allocation, whereas the case managers and allied health professionals responsible for assessment and intervention delivery will be aware of the group assignments owing to the nature of the assessment/intervention.

The intervention group will receive structured ICFIDCM, which includes collaborative goal setting, monthly interdisciplinary case conferences, and coordinated care planning on the basis of ICF assessments. The control group will continue to receive usual multidisciplinary care involving standard discipline-specific assessments and interventions without structured interdisciplinary case management. During the six-month period, participants are permitted to continue all routine medical services and medications that participants are already receiving at baseline. The case managers will contact the case regularly via phone for check-in or service arrangements. If a participant is admitted to the hospital for new medical treatment or if their condition changes significantly, the case manager will notify the SI. Such events and any subsequent change in the intervention or usual care delivery will be recorded as protocol deviations.

**Figure 1:**
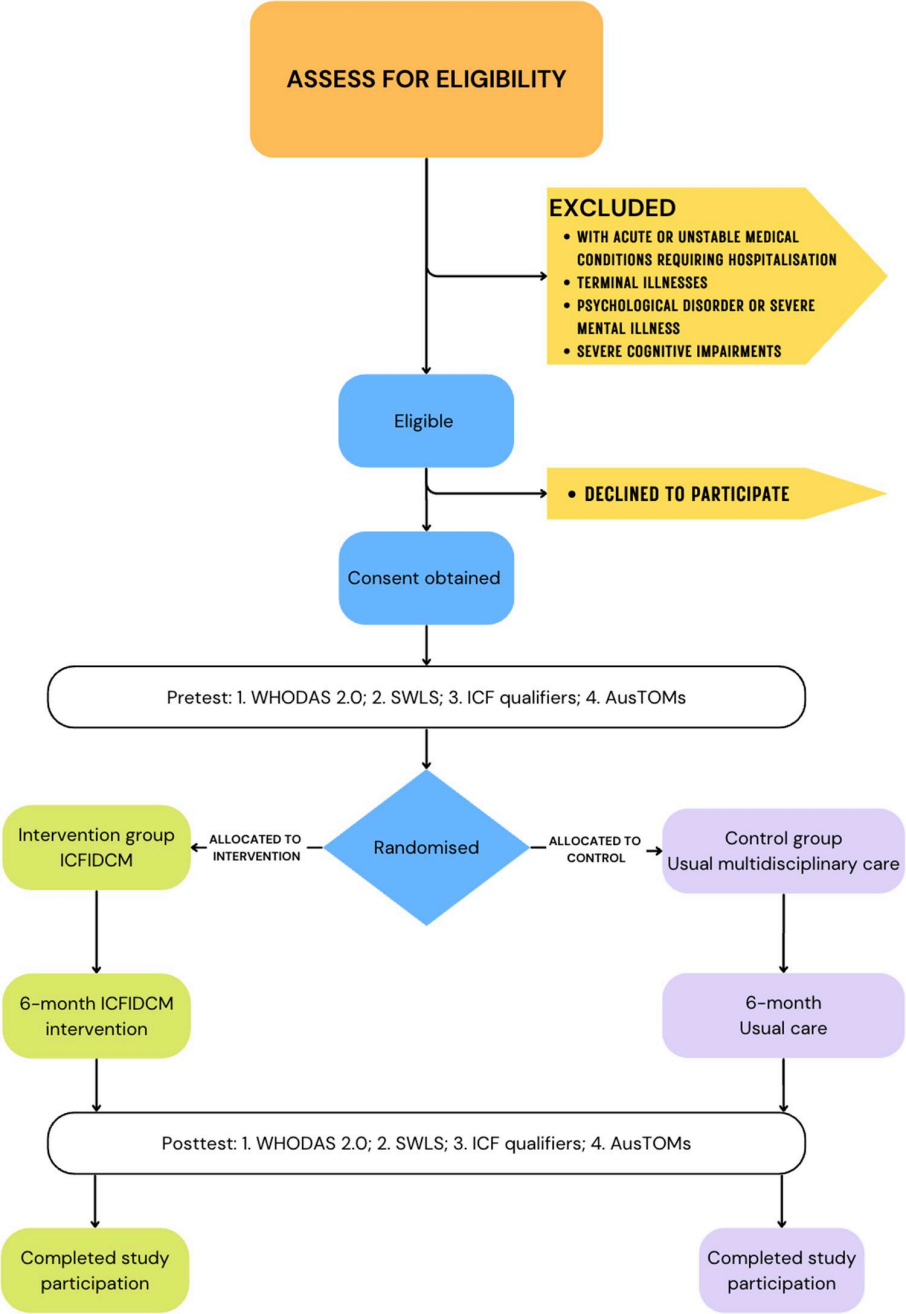
Flow diagram: study design and data collection procedures. ICFIDCM: International Classification of Functioning, Disability and Health-based interdisciplinary case management; WHODAS 2.0: World Health Organization Disability Assessment Schedule 2.0; SWLS: Satisfaction with Life Scale; ICF: International Classification of Functioning, Disability and Health; AusTOMs: Australian Therapy Outcome Measures.

All outcome assessments will be repeated at six months post-intervention. Throughout the study, participants' data and outcomes will be securely stored on an encrypted, password-protected server. Only authorised study personnel will have access, and data will be anonymised using the unique case referral number as the study code. Personal identifiers in documents are stored in locked filing cabinets to maintain confidentiality (Figure 1).

### 2.8 Intervention

Both intervention and control groups are served by the same interdisciplinary team of social workers, physiotherapists, occupational therapists, speech therapist, and nurses. Professional contacts will be individualised based on participants' clinical and functional needs, with frequency, duration, and intensity determined by routine service criteria. Since both groups are delivered in the same service setting and resource structure, protocol-driven differences in treatment intensity or overall service provision are not anticipated. All professional contacts will be systematically recorded. The primary distinction between groups lies in the care coordination structure, as detailed in Sections 2.8.1, 2.8.2.

#### 2.8.1 Control group: usual multidisciplinary case management

The participants in the control group will receive conventional multidisciplinary rehabilitation services, where different professionals (e.g., social workers, physiotherapists, occupational therapists, speech therapist, and nurses) work independently in parallel with the limited integration of care plans. Rehabilitation goals are typically discipline-specific and reported during regular case conferences, whereas coordination among team members occurs on an as-needed basis (22).

#### 2.8.2 Intervention group: ICFIDCM

The participants will receive an ICFIDCM programme, which involves collaborative assessment, interdisciplinary goal settings, and regular case conferences by an interdisciplinary team involving a social worker, physiotherapist, occupational therapist, nurse and speech therapist. To facilitate communication among different professionals, we adopt the conceptual framework of the ICF. This classification is based on two parts. Part one (functioning and disability) consists of 3 components: body functions and structures, activities, and participation. Part two (contextual factors) consists of 2 components: environmental and personal factors. The ICF provides a common language for describing the health states, outcomes and needs of clients, which improves communication between different professionals (24). In addition, it emphasises addressing not only functional impairments, but also environmental and personal factors that influence independence, participation, and overall well-being.

To apply the ICF framework more efficiently, specific core sets, the ICF Rehabilitation Set (also known as the Generic-30 Set) and the Minimal Set of Environmental Factors, with selected categories from the entire ICF category, are adopted to ensure that the relevant functions of people with disabilities are being addressed (26). For each of the selected categories, we will use ICF qualifiers to record the extent of the “problem” with respect to impairment, activity limitation and participation. ICF qualifiers are numeric measures coded after the relevant category code, which are essential to the meaningful use of the ICF classification (Table 1). For categories related to activities and participation domains, ICF qualifiers rate both performance, which describes what an individual does in their current environment, and capacity, which describes an individual's ability to execute a task or an action in a standardised environment.

**Table 1:** Generic ICF qualifiers.

Code	Description
.0	No problem
.1	Mild problem
.2	Moderate problem
.3	Severe problem
.4	Complete problem
.8	Not specified
.9	Not applicable

For activities and participation, qualifiers are applied to both *Performance* and *Capacity*.

The environmental factors qualifiers indicate the extent to which an environmental factor acts as either a facilitator or a barrier. (Table 2).

**Table 2:** ICF environmental factors qualifiers.

Code	Barrier (.)	Code	Facilitator (+)
.0	No barrier	+0	No facilitator
.1	Mild barrier	+1	Mild facilitator
.2	Moderate barrier	+2	Moderate facilitator
.3	Severe barrier	+3	Substantial facilitator
.4	Complete barrier	+4	Complete facilitator
.8	Barrier, not specified	+8	Facilitator, not specific
.9	Not applicable	+9	Not applicable

To gather information to rate the extent of impairment, limitations and restrictions, we will assess the health status of participants based on basis fo the different domains of the ICF (43, 44). These include body functions and structure through, e.g., BMI, pain, and mobility level; activities and participation through the Barthel index; modified functional ambulation classification; the Lawton Instrumental Activities of Daily Living Scale; and environmental and personal factors through, e.g., medications, the use of assistive devices, and family support.

During the initial assessment, similar to the usual care group, the participants (and their caregivers, if any) discuss their treatment goal(s) with the team. The goal-setting interview begins with the case manager, who is the social worker within the team, asking guiding questions on participants' life roles, challenges with daily routines and participation in the community, and then short- and long-term goals. The priorities and ways to integrate these goals into rehabilitation services are also discussed.

The first interdisciplinary case conference will be held after the initial assessment. The interdisciplinary team exchanges information, addressing the health status, identified functioning and contextual factors related to individual goals of rehabilitation. With the contribution of each professional staff member, common care goals addressing the needs of the individual to reintegrate into the community will be set, and an individualised care plan will be formulated. The goals of rehabilitation and related training contents, intensities, durations, completion criteria and responsible professionals will be set and discussed. The responsible case manager of each participant will oversee and integrate services across the interdisciplinary teams. Usually, the first case conference for each participant lasts for 45 min to 1 h. Afterwards, monthly case conferences will be held to review progress and adjust goals and interventions as needed, which usually take approximately 20–30 min per participant.

### 2.9 Outcome measures

All participants will be assessed at baseline (preintervention) and after six months (postintervention) via the following assessment tools.

#### 2.9.1 Primary outcomes

##### 2.9.1.1 Assessment of functional and participation status

The 36-item self-administered version of the WHODAS 2.0 was used to assess functioning and participation across six domains: cognition, mobility, self-care, getting along, life activities, and participation. Participants rated their difficulty experienced in performing activities over the past 30 days on a 5-point Likert scale ranging from 1 (none) to 5 (extreme/cannot do). Domain and summary scores were transformed to a score range from 0 to 100, with higher scores indicating greater disability. Extensive research in 14 countries has shown the strong clinometric properties of the WHODAS 2.0, including internal consistency, reliability, validity, and responsiveness (45).

WHODAS was initially disseminated as WHODAS II, a 36-item instrument grounded in the ICF components of activity and participation, and was subsequently standardised in the 2010 WHO Manual as WHODAS 2.0, which superseded the earlier version while retaining the six-domain, 36-item structure (46). The present study adopted the validated Traditional Chinese self-administered version developed in Hong Kong (WHODAS II CT) (47). This version uses the same item content, response options, recall period, and scoring algorithm as the original 36-item schedule and is therefore equivalent in format and scoring to the WHODAS 2.0 36-item self-administered version described in the WHO manual. The Hong Kong validated study reported excellent internal consistency (Cronbach's *α* 0.89–0.98; overall *α* = 0.98), satisfactory construct validity (*χ*²/df = 3.05, Comparative fit index = 0.912), and good convergent validity in persons with disabilities and chronic illnesses, supporting its appropriateness for use in the present study.

##### 2.9.1.2 Assessment of well-being by participants

The Chinese version of the SWLS (48) will be used to measure life satisfaction. This scale is a short five-item (e.g., “I am satisfied with my life”) scale based on a seven-point rating scale that ranges from 1 (strongly disagree) to 7 (strongly agree). The SWLS has demonstrated a good reliability coefficient of.82 (46) and has been employed in previous research using Chinese samples (*α* = .84) (49).

#### 2.9.2 Secondary outcomes

##### 2.9.2.1 Assessment of functional status by professionals

Score of ICF qualifiers: ICF qualifiers will be used to assess the baseline functional status of each selected category in the ICF Rehabilitation Set and Minimal Set of Environmental Factors. The score of each category is based on observed or self-reported data. The qualifier will be reassessed during postintervention assessment to record any changes in functional status (50). Previous studies have demonstrated the clinical utility, feasibility and inter-rater reliability of ICF qualifiers when applied with structured ICF core sets in rehabilitation settings (12, 13). The ICF Rehabilitation Set has shown acceptable content validity and feasibility in clinical validation studies, supporting its structured use for documenting functional status over time (26, 50).

##### 2.9.1.2 Assessment of participation and well-being by professionals

AusTOMs: Developed by Unsworth & Duncombe (51), AusTOMs measure therapy outcomes for professionals, including occupational therapists, physiotherapists and speech pathologists. The scale, which is based on the WHO concept, contains 4 domains, which are impairment, activity limitation, participation restriction and distress. The Impairment domain describes structural (anatomical) or functional (physiological or psychological) difficulties that a client may have. The activity limitation domain measures a client's level of ability and difficulty in performing activities. The participation restriction domain examines, overall, the limitations that a client may experience in real-life, daily situations. The Distress/Wellbeing domain describes clients' and carers' level of concern. Concern may be evidenced by anger, frustration, apathy or depression. Abu-Awad et al. (51) confirmed the scale's concurrent validity and inter- and intrarater reliability.

## 3 Data analysis

Data analysis will begin only after the follow-up stage. The PI and SI will have access to the final trial dataset. The quantitative data will be analysed via SPSS Statistics 27.0 (IBM). Data cleaning will include systematic screening for outliers and any inconsistencies in the data. Multiple imputations will be considered for missing data. Descriptive statistics will be applied as appropriate for all variables.

**Primary outcomes.** To determine whether functioning (WHODAS 2.0) and subjective well-being (SWLS) improve following the implementation of the ICFIDCM, we will compare intervention and control groups via generalised linear mixed models (GLMMs). Fixed effects will include time (baseline, 6 months), study group, and the group × time interaction, with participant ID as a random effect to account for repeated measures. The target test statistic will be the mean difference (with 95% confidence intervals), estimated via maximum likelihood methods. Two-sided significance will be set at 0.05. **Secondary outcomes.** To analyse the target secondary outcomes (ICF qualifiers and AusTOMs) over the course of time, descriptive analyses will first summarise change scores over time. Subsequently, GLMMs will be used to estimate group differences in changes, with the same fixed- and random-effects structure as above. The results will be expressed as estimated marginal means with 95% confidence intervals.

Treatment dosage variables (e.g., number of visits, total contact time) will be recorded and included as covariates in exploratory sensitivity analyses to assess their potential influence on treatment effects. Although the study is not powered for formal subgroup comparisons, exploratory sensitivity analyses will be performed to examine potential effect modification. Specifically, interaction terms between group allocation and age group, primary diagnosis, and treatment dosage will be examined within the mixed-effects model framework. These analyses are exploratory in nature and will be interpreted cautiously given the limited sample size.

## 4 Discussion

This RCT protocol is designed to evaluate the effectiveness of an ICFIDCM model in enhancing functioning, participation, and well-being among PWD receiving community rehabilitation. By systematically integrating the ICF framework into assessment, goal setting, and care coordination, this study addresses persistent gaps in CDR practice and research, particularly the need for holistic, person-centred approaches that move beyond impairment-focused care.

The anticipated impact of this study is significant. First, it provides empirical evidence of the value of a comprehensive, biopsychosocial model that incorporates not only impairments and activities but also participation and contextual factors—central tenets of the ICF. This approach aligns with international recommendations for disability services that are both evidence-based and responsive to the lived realities of PWD, supporting a shift towards integrated, person-centred care (52, 53). Systematic reviews have shown that integrated biopsychosocial and person-centred rehabilitation approaches improve functional outcomes, participation, and quality of life for people with disability and chronic disease (54, 55).

Second, by directly comparing an ICFIDCM model to traditional multidisciplinary care, this study will clarify whether structured collaboration and coordinated planning lead to superior outcomes in terms of functioning, participation, and subjective well-being, outcomes that are increasingly recognised as critical indicators of successful rehabilitation (56). Integrated care models that enhance interprofessional collaboration and coordinated care planning have been shown to reduce the fragmentation of services and improve PROs across multiple health domains (57, 58).

Third, the findings of this trial may have important implications for service planning and policy development in CDR. Should the ICFIDCM prove effective, it could serve as a scalable, practical framework for improving the quality and consistency of the CDR model. This would support global priorities under WHO's Rehabilitation 2030 initiative, which calls for the integration of ICF within rehabilitation and health systems and promotes health equity and social inclusion (59). This is particularly relevant given the growing evidence that case management models, when delivered by multidisciplinary teams and incorporating social work and holistic planning, can increase patient satisfaction and improve self-reported health status, even if effects on health care utilisation and costs are variable (60, 61). Furthermore, building on the contextual findings of the ICF core set in Hong Kong (32), this trial will expand the evidence base by evaluating the practical implementation and outcomes of an ICFIDCM guided by the ICF core set in community rehabilitation.

The broader public health mission involves strengthening CDR and long-term care systems that support people living with disabilities (59, 62). The ICFIDCM enables this trial to develop rehabilitation practices that are evidence-informed, person-centred, and equitable. The research results will help inform new policy planning, funding systems, and workforce development that support disability-inclusive health systems based on the biopsychosocial model.

### 4.1 Limitations

Despite these strengths, several limitations must be acknowledged. As a single-blind study, although self-reported outcome measures are collected by research assistants blinded to group allocation, some risk of bias remains for therapist-rated outcomes. Additionally, the study focuses primarily on environmental factors within the ICF framework; the operationalisation and measurement of personal factors, such as motivation and coping, remain limited owing to the lack of standardised tools. This highlights an important area for future research.

Another consideration is participant recruitment and retention. As with many community-based intervention studies, there is a possibility of selective participation, with those who are more motivated or engaged being more likely to enrol and complete the study. To mitigate this, sample size calculations account for potential attrition, and efforts will be made to support continued participation throughout the intervention period.

Importantly, while this study will systematically assess environmental factors via the ICF framework, it may not fully capture the breadth and complexity of personal factors, such as motivation and coping, owing to current limitations in standardised measurement tools for these domains (12, 46). This limitation highlights an important area for future research.

While the current trial focuses on clinical effectiveness outcomes, future research should include a dedicated economic evaluation to assess the cost-effectiveness of the ICFICDM model. However, a formal cost-effectiveness analysis requires predefined cost perspectives, systematic economic data collection, and appropriate methodological design, which are beyond the scope of this study. Nevertheless, the clinical effectiveness data generated from the present trial will provide a necessary foundation for such future economic evaluations.

## 5 Ethics and dissemination

### 5.1 Ethics approval and consent to participate

All study procedures were examined and approved by the Research Ethics Committee of the Hong Kong Metropolitan University (reference number: HE–SF2025/27). Only the PI and SI will handle all the information obtained for research purposes. Each participant's data will be anonymised by assigning a unique code. Identifying information will be stored separately in an encrypted file accessible only to authorised research team members. Hard copies, such as consent forms, will be secured in locked cabinets, whereas digital records will be encrypted and stored on a password-protected server, with only the research team having access. Data will be analysed in aggregate form to ensure confidentiality. In compliance with the organisation's service quality standard (YMMSS), data will be stored for three years post-study. After this period, physical records will be shredded, and digital files will be permanently deleted. Any significant modifications to research protocols, adverse events and other unintended effects of trial interventions will be directly reported to the above Research Ethics Committee.

### 5.2 Dissemination

Participant recruitment commenced on 27 May 2025 and will continue until 31 December 2025. Data collection and follow-up are expected to be completed by 31 July 2026. The results of the study are anticipated to be submitted for publication by the end of 2026. The research findings will be presented at local and international conferences for public dissemination and published in peer-reviewed journals. Following the study's completion, the full protocol, participant-level dataset, and statistical code will be made publicly available.

## 6 Conclusion

In summary, this study is positioned to make a significant contribution to the evidence base for the ICFIDCM in CDR. By evaluating both outcomes and implementation processes, this research aims to inform best practices, support policy innovation, and ultimately enhance the quality of life for PWD receiving community-based care. The findings of this study will contribute to advancing evidence-informed, integrated rehabilitation models that more effectively address the multidimensional needs of PWD.

## Data Availability

The original contributions presented in the study are included in the article/Supplementary Material, further inquiries can be directed to the corresponding authors.

## References

[1] Yin YN Wang Y Jiang NJ Long DR. Can case management improve cancer patients quality of life?: a systematic review following PRISMA. Medicine (United States). (2020) 99:e22448. 10.1097/MD.0000000000022448 PMC7535784 33019431

[2] Xin W Xu D Dou Z Jacques A Umbella J Hill AM. Effectiveness of community-based rehabilitation (CBR) centers for improving physical fitness for community-dwelling older adults: a systematic review and meta-analysis. Ann Rehabil Med. (2024) 48:5–21. 10.5535/arm.23148 38433005 PMC10915308

[3] Wijekoon A Jayawardana S Milton-Cole R Chandrathilaka M Jones A Cook S Effectiveness and equity in community-based rehabilitation on pain, physical function, and quality of life after unilateral lower limb amputation: a systematic review. Arch Phys Med Rehabil. (2023) 104:1484–97. 10.1016/j.apmr.2023.02.009 36893877

[4] WHO. Regional Strategic Framework on Community-Based Rehabilitation (CBR) in the South-East Asia Region 2012–2017. Available online at: https://www.who.int/publications/i/item/sea-disability-4 (Accessed September 17, 2025).

[5] WHO. Community Based Rehabilitation Matrix. Community-Based Rehabilitation: CBR Guidelines. 2010. Available online at: https://www.who.int/publications/i/item/9789241548052 (Accessed September 17, 2025).

[6] De Groote W. Concept changes and standardizing tools in community-based rehabilitation. Phys Med Rehabil Clin N Am. (2019) 30:709–21. 10.1016/j.pmr.2019.07.013 31563164

[7] World Health Organisation. International Classification of Functioning Disability and Health (ICF). Geneva: World Health Organization (2001).

[8] Mitchell GK Brown RM Erikssen L Tieman JJ. Multidisciplinary care planning in the primary care management of completed stroke: a systematic review. BMC Fam Pract. (2008) 9:44. 10.1186/1471-2296-9-44 18681977 PMC2518150

[9] Drake RE Green AI Mueser KT Goldman HH. The history of community mental health treatment and rehabilitation for persons with severe mental illness. Community Ment Health J. (2003) 39:427–40. 10.1023/A:1025860919277 14635985

[10] Kim W Ahn MR Kim S Lee E Lee MJ Kim MS. Function and environmental factors analysis using ICF (international classification of functioning, disability and health) for people with disabilities. Ann Rehabil Med. (2008) 32:100–5.

[11] Lee CY Lai HY Lee CH Chen MM Yau SY. Collaborative clinical reasoning: a scoping review. PeerJ. (2024) 12:e17042. 10.7717/peerj.17042 38464754 PMC10924455

[12] Cerniauskaite M Quintas R Boldt C Raggi A Cieza A Bickenbach JE Systematic literature review on ICF from 2001 to 2009: its use, implementation and operationalisation. Disabil Rehabil. (2011) 33:281–309. 10.3109/09638288.2010.529235 21073361

[13] Rauch A Cieza A Stucki G. How to apply the international classification of functioning, disability and health (ICF) for rehabilitation management in clinical practice. Eur J Phys Rehabil Med. (2008) 44:329–42. 18762742

[14] Allan CM Campbell WN Guptill CA Stephenson FF Campbell KE. A conceptual model for interprofessional education: the international classification of functioning, disability and health (ICF). J Interprof Care. (2006) 20:235–45. 10.1080/13561820600718139 16777791

[15] Dan B. The ICF as a socio-psycho-biological model for the full participation of disabled individuals. Dev Med Child Neurol. (2024) 66:1398–9. 10.1111/dmcn.16044 39094060

[16] Sabariego C Barrera AE Neubert S Stier-Jarmer M Bostan C Cieza A. Evaluation of an ICF-based patient education programme for stroke patients: a randomized, single-blinded, controlled, multicentre trial of the effects on self-efficacy, life satisfaction and functioning. Br J Health Psychol. (2013) 18:707–28. 10.1111/bjhp.12013 23252844

[17] Rabea BM Obaidul HM. The use of ICF in physiotherapy management for patient with ischemic stroke: a case study. J Physiother Rehabil. (2019) 2:2.

[18] Wong MNK Cheung MKT Ng YM Yuan HL Lam BYH Fu SN International classification of functioning, disability, and health-based rehabilitation program promotes activity and participation of post-stroke patients. Front Neurol. (2023) 14:1235500. 10.3389/fneur.2023.1235500 38020626 PMC10657202

[19] Körner M. Interprofessional teamwork in medical rehabilitation: a comparison of multidisciplinary and interdisciplinary team approach. Clin Rehabil. (2010) 24:745–55. 10.1177/0269215510367538 20530646

[20] Markle-Reid M Browne G Gafni A Roberts J Weir R Thabane L The effects and costs of a multifactorial and interdisciplinary team approach to falls prevention for older home care clients “at risk” for falling: a randomized controlled trial. Can J Aging. (2010) 29:139–61. 10.1017/S0714980809990377 20202271

[21] Stucki G Cieza A Ewert T Kostanjsek N Chatterji S ÜstÜn TB. Application of the international classification of functioning, disability and health (ICF) in clinical practice. Disabil Rehabil. (2002) 24:281–2. 10.1080/09638280110105222 12004974

[22] Momsen AM Rasmussen JO Nielsen CV Iversen MD Lund H. Multidisciplinary team care in rehabilitation: an overview of reviews. J Rehabil Med. (2012) 44:901–12. 10.2340/16501977-1040 23026978

[23] Grandisson M Hébert M Thibeault R. A systematic review on how to conduct evaluations in community-based rehabilitation. Disabil Rehabil. (2014) 36:265–75. 10.3109/09638288.2013.785602 23614357 PMC3913006

[24] Rentsch HP Bucher P Dommen Nyffeler I Wolf C Hefti H Fluri E The implementation of the “international classification of functioning, disability and health” (ICF) in daily practice of neurorehabilitation: an interdisciplinary project at the Kantonsspital of Lucerne, Switzerland. Disabil Rehabil. (2003) 25:411–21. 10.1080/0963828031000069717 12745951

[25] Leonardi M Fheodoroff K. Goal setting with ICF (international classification of functioning, disability and health) and multidisciplinary team approach in stroke rehabilitation. In: Platz T, editor. Clinical Pathways in Stroke Rehabilitation: Evidence-based Clinical Practice Recommendations. Cham (CH): Springer (2021). p. 35–56. 36315695

[26] Prodinger B Cieza A Oberhauser C Bickenbach J Üstün TB Chatterji S Toward the international classification of functioning, disability and health (ICF) rehabilitation set: a minimal generic set of domains for rehabilitation as a health strategy. Arch Phys Med Rehabil. (2016) 97:875–84. 10.1016/j.apmr.2015.12.030 26827829

[27] Ngangue PA Forgues C Nguyen T Sasseville M Gallagher F Loignon C Patients, caregivers and health-care professionals’ experience with an interdisciplinary intervention for people with multimorbidity in primary care: a qualitative study. Health Expect. (2020) 23:318–27. 10.1111/hex.13035 32035012 PMC7104629

[28] Birkeland A Tuntland H Førland O Jakobsen F Langeland E. Interdisciplinary collaboration in reablement-a qualitative study. J Multidiscip Healthc. (2017) 10:195–203. 10.2147/JMDH.S133417 28503067 PMC5426462

[29] Madden RH Bundy A. The ICF has made a difference to functioning and disability measurement and statistics. Disabil Rehabil. (2019) 41:1450–62. 10.1080/09638288.2018.1431812 29433362

[30] Leonardi M Lee H Kostanjsek N Fornari A Raggi A Martinuzzi A 20 years of ICF—international classification of functioning, disability and health: uses and applications around the world. Int J Environ Res Public Health. (2022) 19:11321. 10.3390/ijerph191811321 36141593 PMC9517056

[31] Hernández-Lázaro H Mingo-Gómez MT Jiménez-del-Barrio S Simarro-Martín A Wiśniowska-Szurlej A Ceballos-Laita L. Development of an ICF core set for the management of musculoskeletal conditions in primary care physiotherapy services in Spain: a Delphi study. Disabil Rehabil. (2025) 47:4759–68. 10.1080/09638288.2025.2460723 39931756

[32] Yeung CK Wong PK Cheng KM Wong SS Cheng HY Cheung WY Development of the international classification of functioning, disability and health (ICF) core set for stroke survivors in community-based rehabilitation settings in Hong Kong. Disabil Rehabil. (2025):1–15. 10.1080/09638288.2025.2557499 40924797

[33] Abarghuei AF Mehraban AH Lajevardi L Yousefi M. The clinical application of ICF model for occupational therapy in a patient with stroke: a case report. Med J Islam Repub Iran. (2018) 32:65.30643740

[34] Wong MNK Tong H Cheung MKT Ng YM Yuan HL Lam BYH Goal-setting and personalization under the international classification of functioning: disability, and health framework: Community reintegration program for post-stroke patients. Front Rehabil Sci. (2023) 4:1219662. 10.3389/fresc.2023.1219662 37600161 PMC10436562

[35] Hussain B Khalily MT Waqas A Rahman A Angelakis I Nisar A Acceptability and efficacy of the culturally adapted problem management plus intervention for people with disability in Pakistan: a pilot cluster randomized controlled trial. Front Psychiatry. (2025) 15:1413809. 10.3389/fpsyt.2024.1413809 39980593 PMC11841386

[36] Faul F Erdfelder E Lang AG Buchner A. G*Power 3: a flexible statistical power analysis program for the social, behavioral, and biomedical sciences. Behav Res Methods. (2007) 39:175–91. 10.3758/BF03193146 17695343

[37] Efird J. Blocked randomization with randomly selected block sizes. Int J Environ Res Public Health. (2011) 8:15–20. 10.3390/ijerph8010015 21318011 PMC3037057

[38] Yabuwaki K Shinohara K Fujioka A Inagaki S Hirao K. Effectiveness of comprehensive environmental support for community-dwelling older adults: a single-blind randomized controlled trial. Am J Occup Ther. (2024) 78:7803205070. 10.5014/ajot.2024.050431 38602705

[39] Verghese J Mahoney JR Ayers E Ambrose A Wang C Holtzer R. Computerised cognitive remediation to enhance mobility in older adults: a single-blind, single-centre, randomised trial. Lancet Healthy Longev. (2021) 2:e571–9. 10.1016/S2666-7568(21)00173-2 34522910 PMC8437150

[40] Edwards P. Questionnaires in clinical trials: guidelines for optimal design and administration. Trials. (2010) 11:2. 10.1186/1745-6215-11-2 20064225 PMC2823735

[41] Karanicolas PJ Farrokhyar F Bhandari M. Blinding: who, what, when, why, how? Can J Surg. (2010) 53:345–8. 20858381 PMC2947122

[42] Stuart-Hamilton I. Single-Blind study. In: Salkind NJ, editor. Encyclopedia of Research Design. Thousand Oaks, CA: SAGE Publications, Inc (2010). p. 1384–6. doi: 10.4135/9781412961288.n423.

[43] Noohu MM Dey AB Sharma S Hussain ME. International classification of function, disability and health framework for fall risk stratification in community dwelling older adults. Geriatric Care (Pavia). (2017) 3:e6526. 10.4081/gc.2017.6526

[44] Soh SE Barker AL Morello RT Ackerman IN. Applying the international classification of functioning, disability and health framework to determine the predictors of falls and fractures in people with osteoarthritis or at high risk of developing osteoarthritis: data from the osteoarthritis initiative. BMC Musculoskelet Disord. (2020) 21(1):138. 10.1186/s12891-020-3160-5 32113478 PMC7049177

[45] Bejer A Ćwirlej-Sozańska A Wiśniowska-Szurlej A Wilmowska-Pietruszyńska A Spalek R de Sire A Psychometric properties of the polish version of the 36-item WHODAS 2.0 in patients with hip and knee osteoarthritis. Qual Life Res. (2021) 30:2415–27. 10.1007/s11136-021-02806-4 33719013 PMC8298349

[46] Üstün T Kostanjsek N Chatterji S Rehm J. Measuring Health and Disability Manual for WHO Disability Assessment Schedule WHODAS 2.0 (2010). World Health Organization. Available online at: https://www.who.int/publications/i/item/measuring-health-and-disability-manual-for-who-disability-assessment-schedule-(-whodas-2.0) (Accessed September 17, 2025).

[47] Cheung MKT Hung ATF Poon PKK Fong DYT Li LSW Chow ESL Validation of the world health organization assessment schedule II Chinese traditional version (WHODAS II CT) in persons with disabilities and chronic illnesses for Chinese population. Disabil Rehabil. (2015) 37:1902–7. 10.3109/09638288.2014.989336 25495681

[48] Sachs J. Psychometric properties of the proactive attitude scale in students at the university of Hong Kong. Psychol Rep. (2003) 93:805–15. 10.2466/pr0.2003.93.3.805 14723447

[49] Chen LH Kee YH. Gratitude and adolescent athletes’ well-being. Soc Indic Res. (2008) 89:361–73. 10.1007/s11205-008-9237-4

[50] Wiśniowska-Szurlej A Sozańska A Brożonowicz J Wilmowska-Pietruszyńska A Sozański B. Validation and evaluation of the application of the ICF rehabilitation set: a Polish clinical perspective. BMC Health Serv Res. (2025) 25:1027. 10.1186/s12913-025-13048-2 40764554 PMC12326788

[51] Abu-Awad Y Unsworth CA Coulson M Sarigiannis M. Using the Australian therapy outcome measures for occupational therapy (AusTOMs-OT) to measure client participation outcomes. Br J Occup Ther. (2014) 77:44–9. 10.4276/030802214X13916969446958

[52] Dutra FCMS Mancini MC Neves JA Kirkwood RN Sampaio RF. Empirical analysis of the international classification of functioning, disability and health (ICF) using structural equation modeling. Braz J Phys Ther. (2016) 20:384. 10.1590/bjpt-rbf.2014.0168 27878225 PMC5123257

[53] Carlin L McPherson G Davison R. The international classification of functioning disability and health framework (ICF): a new approach to enhance sport and physical activity participation among people with disabilities in Scotland. Front Sports Act Living. (2024) 6:1228198. 10.3389/fspor.2024.1225198 PMC10978736 38558859

[54] Valentijn PP Tymchenko L Gruisen W Bruls B Pereira FA Arends RY. Effectiveness of integrated care for diabetes mellitus type 2, cardiovascular and chronic respiratory diseases: a systematic review and meta-analysis. Int J Integr Care. (2024) 24:16. 10.5334/ijic.7744 39184531 PMC11342834

[55] Veroniki AA Soobiah C Nincic V Lai Y Rios P MacDonald H Efficacy of sustained knowledge translation (KT) interventions in chronic disease management in older adults: systematic review and meta-analysis of complex interventions. BMC Med. (2023) 21:1–20. 10.1186/s12916-023-02966-9 37488589 PMC10367354

[56] Albanesi F Invernizzi V Meucci P Leonardi M Caspani G Pessina A Role of disability-case manager for chronic diseases: using the ICF as a practical background ICF as a background. Disabil Rehabil. (2009) 31:S50–4. 10.3109/09638280903317799 19968535

[57] Mitchell GK Burridge L Zhang J Donald M Scott IA Dart J Systematic review of integrated models of health care delivered at the primary-secondary interface: how effective is it and what determines effectiveness? Aust J Prim Health. (2015) 21:391–408. 10.1071/PY14172 26329878

[58] Otieno P Agyemang C Wao H Wambiya E Ng’oda M Mwanga D Effectiveness of integrated chronic care models for cardiometabolic multimorbidity in sub-Saharan Africa: a systematic review and meta-analysis. BMJ Open. (2023) 13:e073652. 10.1136/bmjopen-2023-073652 37369405 PMC10410889

[59] World Health Organization. Rehabilitation 2030: A Call for Action. World Health Organization (2017). Available online at: https://cdn.who.int/media/docs/default-source/documents/health-topics/rehabilitation/call-for-action/rehab2030meetingreport_plain_text_version.pdf (Accessed November 6, 2025).

[60] Joo JY Huber DL. Case management effectiveness on health care utilization outcomes: a systematic review of reviews. West J Nurs Res. (2019) 41:111–33. 10.1177/0193945918762135 29542405

[61] Lukersmith S Millington M Salvador-Carulla L. What is case management? A scoping and mapping review. Int J Integr Care. (2016) 16:2. 10.5334/ijic.2477 28413368 PMC5388031

[62] World Health Organization. Strengthening Rehabilitation in Health Systems: Resolution of the World Health Assembly (2022). World Health Assembly Report EB152(10); Available online at: https://apps.who.int/gb/ebwha/pdf_files/EB152/B152(10)-en.pdf (Accessed November 6, 2025).

